# Electrochemical Pretreatment of Chrysocolla for Enhanced Sulfidization and Flotation: Mechanism and Process Optimization

**DOI:** 10.3390/ma19183944

**Published:** 2026-09-17

**Authors:** Gulnara Katkeeva, Zhamila Shaike, Aidarkhan Mukhtar, Karakat Turebekova, Gulmarzhan Burkitseterkyzy, Yerlan Zhunussov, Anuar Zhunussov, Sultan Kabylkanov

**Affiliations:** 1 Zh. Abishev Chemical-Metallurgical Institute, Karaganda 100030, Kazakhstan; katkeeva@mail.ru (G.K.); zhamilya.shayke@mail.ru (Z.S.); kakosh-94@mail.ru (K.T.); gulmarzhan.94@mail.ru (G.B.); 2 Green Chemicals Kazakhstan, Karaganda 100030, Kazakhstan; xx_m9_xx@mail.ru (Y.Z.); anuar.zhunussov@mail.ru (A.Z.); 3 Department of Metallurgy and Mining, K. Zhubanov Aktobe Regional University, Aktobe 030000, Kazakhstan

**Keywords:** oxidized copper ores, malachite, surface modification, interfacial activation, pulp redox conditioning, copper recovery

## Abstract

The flotation of oxidized copper minerals is limited by poor surface reactivity and the insufficient effectiveness of conventional sulfidization. This study investigated 50 Hz alternating current electrochemical pretreatment to enhance the sulfidization and flotation of chrysocolla and a natural oxidized copper ore. Operating parameters, including current density, treatment time, pulp pH, and sodium sulfide dosage, were optimized using a design of experiments approach. Phase transformations were characterized by scanning electron microscopy with energy-dispersive X-ray spectroscopy and X-ray diffraction. Pretreatment with 50 Hz alternating current promoted the surface formation of tenorite (CuO), which was subsequently transformed into covellite (CuS) during sulfidization, providing favorable conditions for subsequent flotation. The optimum conditions for chrysocolla were a current density of 100 A m^−2^, a pretreatment time of 0.5 min, pH 8, a sodium sulfide dosage of 25% of the stoichiometric requirement, and a sulfidization time of 2.5 min. Validation tests on natural oxidized copper ore showed that alternating current pretreatment increased copper recovery from 29.16% to 79.63% and concentrate grade from 12.84 to 25.50 wt.% compared with conventional sulfidization. These findings demonstrate that alternating current electrochemical pretreatment effectively modifies oxidized copper mineral surfaces and enhances their subsequent sulfidization and flotation, providing a promising approach for the beneficiation of refractory oxidized copper ores.

## 1. Introduction

### 1.1. Current State of Oxidized Copper Ore Processing

The global copper industry is facing a gradual depletion of high-grade sulfide ore reserves, accompanied by an increasing reliance on oxidized and mixed copper ores. This trend is associated with the exploitation of deeper ore horizons, the development of oxidation zones, and the growing utilization of low-grade and technogenic mineral resources. As a result, the processing of refractory oxidized copper ores has become an important direction in the development of advanced copper recovery technologies [1,2].

Oxidized copper ores contain a diverse range of copper-bearing minerals, including malachite, azurite, cuprite, tenorite, chrysocolla, brochantite, and other carbonate, oxide, silicate, and hydroxide minerals. Their heterogeneous mineralogical composition strongly influences their technological behavior and complicates copper recovery by conventional beneficiation methods. In particular, differences in mineral reactivity, surface composition, dissolution behavior, and interaction with flotation reagents can result in substantially different flotation responses among individual copper minerals [3,4].

Several technological approaches are currently employed for the processing of oxidized copper ores, including flotation after surface sulfidization, hydrometallurgical treatment based on acid leaching followed by solvent extraction and electrowinning (SX–EW), and combined flotation–hydrometallurgical flowsheets. The selection of an appropriate processing route depends on the mineralogical characteristics of the ore, copper grade, degree of oxidation, presence of clay and gangue minerals, and the overall economic feasibility of the process [5].

Despite the continued development of hydrometallurgical technologies, flotation remains an economically attractive option for the processing of many oxidized and mixed copper ores. Its efficiency, however, depends strongly on the physicochemical state of mineral surfaces and their conditioning prior to interaction with flotation reagents. Consequently, recent research has increasingly focused not only on optimizing reagent regimes but also on developing methods for targeted modification of mineral surfaces before flotation. Among these approaches, electrochemical, ultrasonic, plasma, and other physicochemical pretreatment methods have attracted considerable attention because of their potential to modify surface reactivity and improve the subsequent sulfidization of oxidized copper minerals [6].

Therefore, the development of efficient pretreatment methods capable of modifying the surface properties of oxidized copper minerals represents an important challenge in modern mineral processing. In this context, electrochemical pretreatment is of particular interest because it can alter the physicochemical state of mineral surfaces and potentially improve their interaction with sulfidizing and flotation reagents. Further investigation of this approach is therefore warranted for the development of more effective flotation technologies for refractory oxidized copper ores.

### 1.2. Challenges in the Flotation of Oxidized Copper Minerals

The efficiency of flotation beneficiation of oxidized copper ores is strongly influenced by the physicochemical state of mineral surfaces. Unlike sulfide minerals, which readily interact with sulfhydryl collectors, oxidized copper minerals generally exhibit hydrophilic surface properties that limit their natural floatability [7].

The surfaces of minerals such as malachite, azurite, cuprite, and particularly chrysocolla are characterized by hydroxyl groups, adsorbed water, and various surface species formed during oxidation and dissolution. In aqueous media, these minerals may undergo partial dissolution with the release of copper-containing species into the pulp, resulting in continuous changes in surface chemistry and potentially reducing the effectiveness of flotation reagent adsorption.

To improve the flotation response of oxidized copper minerals, surface sulfidization using sodium sulfide (Na_2_S) or sodium hydrosulfide (NaHS) is conventionally employed. During sulfidization, the reagent interacts with the mineral surface and promotes the formation of copper sulfide species, which can provide favorable sites for the subsequent adsorption of xanthates and other sulfhydryl collectors.

However, the efficiency of sulfidization is highly sensitive to operating conditions. The formation and stability of an active sulfide layer depend on the sulfidizing agent dosage, conditioning time, pulp pH, redox potential, concentration of dissolved species, ore mineralogy, and degree of surface oxidation. Insufficient sulfidization may result in inadequate collector adsorption, whereas excessive sulfidizing agent dosage can promote the formation of hydrophilic sulfur-containing species and adversely affect flotation performance [8].

Additional challenges arise in the flotation of finely disseminated and mixed oxide–sulfide ores containing copper minerals with different surface properties and reactivities. Under such conditions, conventional reagent schemes may not ensure the formation of a sufficiently stable hydrophobic surface layer on all copper-bearing minerals, resulting in lower copper recovery and reduced process selectivity.

Therefore, the effectiveness of conventional sulfidization is strongly dependent on the initial surface state of oxidized copper minerals and the applied reagent conditions. This limitation highlights the need for pretreatment methods capable of modifying mineral surfaces and creating more favorable conditions for subsequent sulfidization and flotation.

### 1.3. Modern Pretreatment Methods Prior to Flotation

The efficiency of oxidized copper mineral flotation is strongly influenced by the condition of mineral surfaces prior to interaction with flotation reagents. Consequently, pretreatment is an important stage of beneficiation because it can modify surface properties, enhance mineral sulfidization, improve collector adsorption, and ultimately increase copper recovery.

Surface sulfidization using sodium sulfide (Na_2_S) or sodium hydrosulfide (NaHS) remains the most widely applied approach for conditioning oxidized copper minerals prior to flotation. However, its effectiveness depends strongly on the mineral surface properties and operating conditions and may not always result in the formation of a uniform and stable sulfide layer.

To overcome these limitations, various approaches have been developed to intensify the pretreatment of oxidized copper minerals. These include combined reagent schemes, activators, complexing agents, surface modifiers, dispersants, and regulation of the chemical composition of the pulp. Such approaches are primarily aimed at increasing surface reactivity, promoting sulfidization, and improving the selectivity of subsequent flotation [9].

In parallel, physical and physicochemical pretreatment methods, including ultrasonic treatment, mechanochemical activation, plasma treatment, microwave irradiation, and electrochemical treatment, have attracted increasing research interest. Unlike conventional reagent conditioning, these methods can modify the physicochemical state of mineral surfaces, influence mineral dissolution and surface species formation, and alter interfacial processes governing flotation behavior.

Among these approaches, electrochemical pretreatment is particularly promising because it enables controlled modification of mineral surface properties and redox conditions without relying exclusively on increased reagent consumption. However, compared with conventional sulfidization and other pretreatment methods, its application to refractory oxidized copper minerals remains insufficiently investigated. This provides a basis for considering electrochemical pretreatment as a separate conditioning stage prior to sulfidization and flotation.

### 1.4. Electrochemical Pretreatment: Current State of the Art

In recent years, electrochemical methods have attracted increasing attention as a means of intensifying mineral processing technologies. Unlike conventional reagent conditioning, electrochemical pretreatment can modify the physicochemical properties of mineral surfaces, regulate the redox conditions of the pulp, and influence interfacial reactions involved in mineral–reagent interactions.

Previous studies have demonstrated that electrochemical treatment can modify the composition and distribution of surface species, alter the electrochemical state of mineral surfaces, and influence the adsorption and transformation of flotation reagents. These observations are consistent with the established principles of mineral surface electrochemistry, according to which interfacial redox reactions influence surface species formation and the adsorption of sulfhydryl collectors, thereby affecting mineral flotation behavior [6,7,8]. Consequently, controlled electrochemical treatment may increase surface reactivity and modify the conditions under which subsequent reagent–mineral interactions occur.

Particular interest has been directed toward the application of electrochemical pretreatment to oxidized copper minerals, whose flotation is commonly hindered by hydrophilic surface properties and the variable effectiveness of conventional sulfidization. Recent studies suggest that combining electrochemical treatment with subsequent sulfidization can modify the surface state of oxidized copper minerals and improve their interaction with sulfhydryl collectors, resulting in enhanced flotation performance [10].

Recent international progress over the past five years (2021–2025) has further emphasized the efficacy of electrochemical regulation of mineral flotation interfacial behavior. Recent investigations demonstrate that precise surface potential control, interfacial redox conditioning, and dynamic pulp potential stabilization significantly enhance surface phase transformations, collector adsorption kinetics, and copper mineral selectivity while avoiding excessive chemical consumption [11,12,13,14]. However, most recent studies focus on direct current (DC) systems or chemical/thermal activation mechanisms. The application of standard 50 Hz industrial frequency AC to rapidly modify refractory oxidized copper surfaces remains inadequately systematically investigated.

Therefore, electrochemical pretreatment represents a promising approach for modifying the surface state of refractory oxidized copper minerals before sulfidization and flotation. Establishing the relationship between electrochemical operating conditions, surface phase transformations, sulfidization behavior, and flotation performance is essential for developing an effective and technologically applicable pretreatment process.

### 1.5. Research Gap

A review of the available literature indicates substantial progress in understanding the sulfidization of oxidized copper minerals, developing flotation reagent schemes, and applying physicochemical methods for mineral surface modification. However, the interaction between electrochemical pretreatment, subsequent sulfidization, and flotation remains insufficiently understood. In particular, the role of electrochemical treatment in modifying the surface phase composition of oxidized copper minerals prior to sulfidization and its subsequent effect on flotation behavior has received limited systematic investigation [15,16,17].

Previous studies have demonstrated that electrochemical treatment can alter the physicochemical and electrochemical properties of mineral surfaces. However, the relationship between electrochemical treatment conditions, surface phase transformations, sulfide-layer formation, and the resulting flotation response of refractory oxidized copper minerals has not been fully established. This is particularly relevant to chrysocolla, whose hydrated silicate structure and surface properties make it considerably more difficult to sulfidize and float than many other oxidized copper minerals.

Furthermore, few studies have systematically combined mineralogical characterization of surface transformations with optimization of electrochemical pretreatment parameters and subsequent validation using a natural oxidized copper ore. The absence of such an integrated approach limits the understanding of how electrochemical surface modification can be translated into improved sulfidization and flotation performance under technologically relevant conditions.

Therefore, a systematic investigation linking electrochemical pretreatment, surface phase transformation, subsequent sulfidization, and flotation performance is still needed. The present study addresses this gap by elucidating the surface transformations induced by alternating-current electrochemical pretreatment of chrysocolla, optimizing the key pretreatment and sulfidization parameters, and validating the resulting flotation response using a natural oxidized copper ore [18,19,20].

### 1.6. Objective of the Study

The objective of this study was to investigate the effect of alternating-current electrochemical pretreatment on the surface properties, sulfidization, and flotation behavior of chrysocolla; elucidate the associated surface phase transformations; and establish optimal operating conditions for the subsequent sulfidization and flotation of oxidized copper minerals. The applicability of the optimized process was further evaluated using a natural oxidized copper ore.

## 2. Materials and Methods

### 2.1. Materials

The study was conducted using a monomineralic chrysocolla fraction and a technological sample of oxidized copper ore from the Udokan deposit.

The monomineralic chrysocolla fraction was obtained from the technological ore sample by hand-picking under a binocular microscope. The purity of the chrysocolla fraction was approximately 90%. The fraction was used to investigate the effects and mechanisms of electrochemical pretreatment, subsequent sulfidization, and flotation under conditions minimizing the influence of associated minerals.

For validation of the proposed technology under conditions representative of a natural multicomponent ore, a technological sample of oxidized copper ore from the Udokan deposit was used. A representative 200 kg sample with an initial particle size of −100 + 0 mm was prepared for the experimental study. The average copper content of the ore was 1.3 wt.%. Mineralogical analysis showed that copper occurred predominantly as oxidized copper minerals, with chrysocolla and malachite identified as the principal copper-bearing phases.

### 2.2. Electrochemical Pretreatment

Electrochemical pretreatment was applied to modify the physicochemical state of the chrysocolla surface prior to subsequent sulfidization and flotation. The treatment was performed by passing an electric current through the mineral pulp, thereby altering the electrochemical conditions at the mineral–solution interface and promoting changes in the surface species relevant to subsequent sulfidization.

Both DC and AC were investigated. For each treatment mode, the effects of electrochemical pretreatment on chrysocolla surface reactivity, xanthate interaction, subsequent sulfidization, and flotation response were evaluated. Alternating current at an industrial frequency of 50 Hz was selected for further optimization based on preliminary experiments that demonstrated a more pronounced improvement in the flotation response of chrysocolla.

The effects of current density, electrochemical treatment time, pulp pH, and sodium sulfide dosage were systematically investigated. The optimum operating conditions were established using a design of experiments (DOE) approach, which allowed the individual and combined effects of the investigated parameters on flotation performance to be evaluated.

Following electrochemical pretreatment, the pulp was subjected to sulfidization with an aqueous sodium sulfide solution and subsequently processed by flotation. Samples collected after electrochemical pretreatment and after sulfidization were characterized by X-ray diffraction (XRD), scanning electron microscopy (SEM), and energy-dispersive X-ray spectroscopy (EDS) to identify surface and phase transformations associated with the treatment sequence.

All flotation tests were conducted using analytically pure reagents to ensure high experimental reproducibility. Sodium sulfide (Na_2_S, >98.0% purity, Sigma-Aldrich, St. Louis, MO, USA) was utilized as the sulfidizing agent. Sodium butyl xanthate (SIBX, >90.0% purity, Fluka, St. Louis, MO, USA) served as the primary sulfhydryl collector. Terpineol (technical grade, Aldrich, St. Louis, MO, USA) was employed as the frother at a constant concentration of 20 mg/L. Micro-analytical grade hydrochloric acid (HCl) and sodium hydroxide (NaOH) were used for pH regulation. Deionized water (resistivity >18.2 M ohm cm) was used in all electrochemical conditioning, slurry preparation, and flotation experiments [21].

To isolate the influence of individual variables during single-factor screening experiments, a fixed baseline parameter set was established. When a specific factor was varied, all other operational parameters were maintained strictly at their baseline values: current density of 100 A/m^2^, AC electrochemical pretreatment time of 0.5 min, AC electric field frequency of 50 Hz, sodium sulfide (Na_2_S) dosage of 25% of the stoichiometric requirement, pulp pH of 8.0, sulfidization time of 2.5 min, collector (SIBX) dosage of 200 g/t, collector conditioning time of 3.0 min, frother (Terpineol) dosage of 20 mg/L, impeller rotation speed of 1500 rpm, airflow rate of 1.5 L/min, and cumulative flotation time of 5.0 min.

### 2.3. Flotation Procedure

Flotation experiments were conducted to evaluate the effect of electrochemical pretreatment on the flotation response of chrysocolla and oxidized copper ore. The flotation procedure consisted of sequential stages of electrochemical pretreatment, sulfidization, collector conditioning, frother addition, and flotation.

Following electrochemical pretreatment under the specified operating conditions, sodium sulfide (Na_2_S) was added to the pulp and conditioned for the predetermined sulfidization time. The flotation collector was then added and conditioned, followed by the addition of the frother and subsequent flotation. The reagent sequence and conditioning conditions were kept consistent for comparative experiments unless the corresponding parameter was intentionally varied within the experimental design.

The effects of current density, electrochemical treatment time, sodium sulfide dosage, pulp pH, and sulfidization time on flotation performance were investigated. The optimum conditions were determined based primarily on copper recovery and concentrate grade, with the separation efficiency considered as an additional performance indicator where applicable.

The effectiveness of electrochemical pretreatment was evaluated by comparing the flotation results obtained after electrochemical treatment with those obtained using conventional sulfidization and flotation without electrical pretreatment. The resulting concentrates and tailings were subjected to chemical and mineralogical characterization to assess copper distribution and changes in the occurrence of copper-bearing phases.

### 2.4. Mineralogical Characterization

The initial mineral samples and the products obtained after electrochemical pretreatment and subsequent sulfidization were characterized using X-ray diffraction (XRD, Rigaku Corporation, Tokyo, Japan) and scanning electron microscopy coupled with energy-dispersive X-ray spectroscopy (SEM/EDS, ZEM20, ZHIYI Instruments, Hangzhou, China).

XRD analysis was performed to identify the crystalline phases present in the samples and to evaluate phase transformations associated with electrochemical pretreatment and subsequent sulfidization.

The surface morphology and local elemental composition of the mineral samples were examined using SEM/EDS. The SEM images were used to characterize changes in surface morphology, while EDS analysis was used to determine the local distribution of major elements and identify copper-containing surface regions. The SEM/EDS data were interpreted using established approaches for the characterization of the structure, morphology, and elemental composition of mineral and metallurgical materials [9,10]. The obtained results were used to analyze the formation of copper-containing surface species and to evaluate their influence on subsequent sulfidization and flotation.

### 2.5. Experimental Design and Statistical Analysis

The optimization of the electrochemical pretreatment and sulfidization parameters was performed using a design of experiments (DOE) approach. The investigated variables included current density, electrochemical treatment time, pulp pH, sodium sulfide dosage, and sulfidization time. The individual and combined effects of these variables on chrysocolla flotation recovery were evaluated.

Regression models were developed to describe the relationships between the investigated process parameters and flotation performance and to determine the optimum operating conditions. All flotation experiments were performed in triplicate, and the reported values represent the arithmetic mean of the experimental results. The relative experimental error did not exceed 2%.

The efficiency of the proposed process was evaluated based on copper recovery, concentrate grade, and the degree of surface sulfidization, together with the phase transformations identified by X-ray diffraction (XRD) and scanning electron microscopy coupled with energy-dispersive X-ray spectroscopy (SEM/EDS). The degree of sulfidization was evaluated from the decrease in the content of oxidized copper after treatment relative to its initial content before sulfidization [22,23].

## 3. Results and Discussion

### 3.1. Effect of Alternating-Current Electrochemical Pretreatment on the Sulfidization and Flotation of Chrysocolla

The effectiveness of AC electrochemical pretreatment was evaluated based on its influence on the subsequent sulfidization and flotation behavior of chrysocolla.

Alternating current was selected for further investigation because preliminary experiments demonstrated that it provided the most favorable combination of flotation performance and practical feasibility compared with DC. The effects of electrochemical treatment were evaluated both before and after subsequent sulfidization, with particular attention to chrysocolla recovery and the formation of xanthate reaction products on the mineral surface.

The flotation and xanthate adsorption results obtained using different electrochemical treatment modes, both in the absence and in the presence of subsequent sulfidization, are presented in Table 1 and Table 2. All flotation experiments were carried out at a constant room temperature of 22 ± 1 °C in a 100 mL laboratory mechanical flotation cell with an impeller speed of 1500 rpm. Quantitative analysis of potassium ethyl xanthate (PEX) adsorption products was conducted using a UV-1800 UV-Vis spectrophotometer (Shimadzu, Kyoto, Japan) at a characteristic wavelength of 301 nm after centrifugal separation of the liquid phase [24,25,26].

Following sulfidization, AC pretreatment again provided the higher chrysocolla recovery, reaching 28.21%, compared with 20.01% after DC treatment. These results indicate that AC pretreatment creates more favorable surface conditions for subsequent sulfidization and interaction with xanthate collectors.

To further evaluate the effect of AC pretreatment, the flotation performance and composition of xanthate reaction products were compared with those obtained without electrochemical treatment. The results are presented in Table 3.

As shown in Table 3, AC electrochemical pretreatment substantially improved the flotation performance of chrysocolla. The copper grade in the concentrate increased from 18.96 to 32.21 wt.%, while chrysocolla recovery increased from 14.62 to 33.55%. At the same time, changes were observed in the composition of xanthate reaction products formed on the mineral surface. The contents of dixanthogen and copper(I) dixanthogenide decreased, whereas the relative content of copper(I) xanthate increased.

The observed improvement in flotation performance can be attributed to changes in the physicochemical state of the chrysocolla surface induced by AC electrochemical pretreatment. Electrochemical treatment may increase the reactivity of surface sites associated with structural defects and microcracks generated during crushing and grinding, thereby facilitating subsequent interaction with sodium sulfide. This promotes the formation of a more favorable sulfide surface layer for xanthate adsorption and increases the hydrophobicity of the mineral surface. Similar effects of electrochemical surface modification on the flotation behavior of copper-bearing minerals have been reported previously [27,28].

Overall, the results demonstrate that AC electrochemical pretreatment provides more favorable conditions for the subsequent sulfidization of chrysocolla and significantly improves its flotation response. Therefore, AC treatment was selected for the subsequent investigation of the mineralogical transformations and optimization of the main process parameters.

### 3.2. Mineralogical Evidence for the Mechanism of Electrochemical Pretreatment

**Figure 1:**
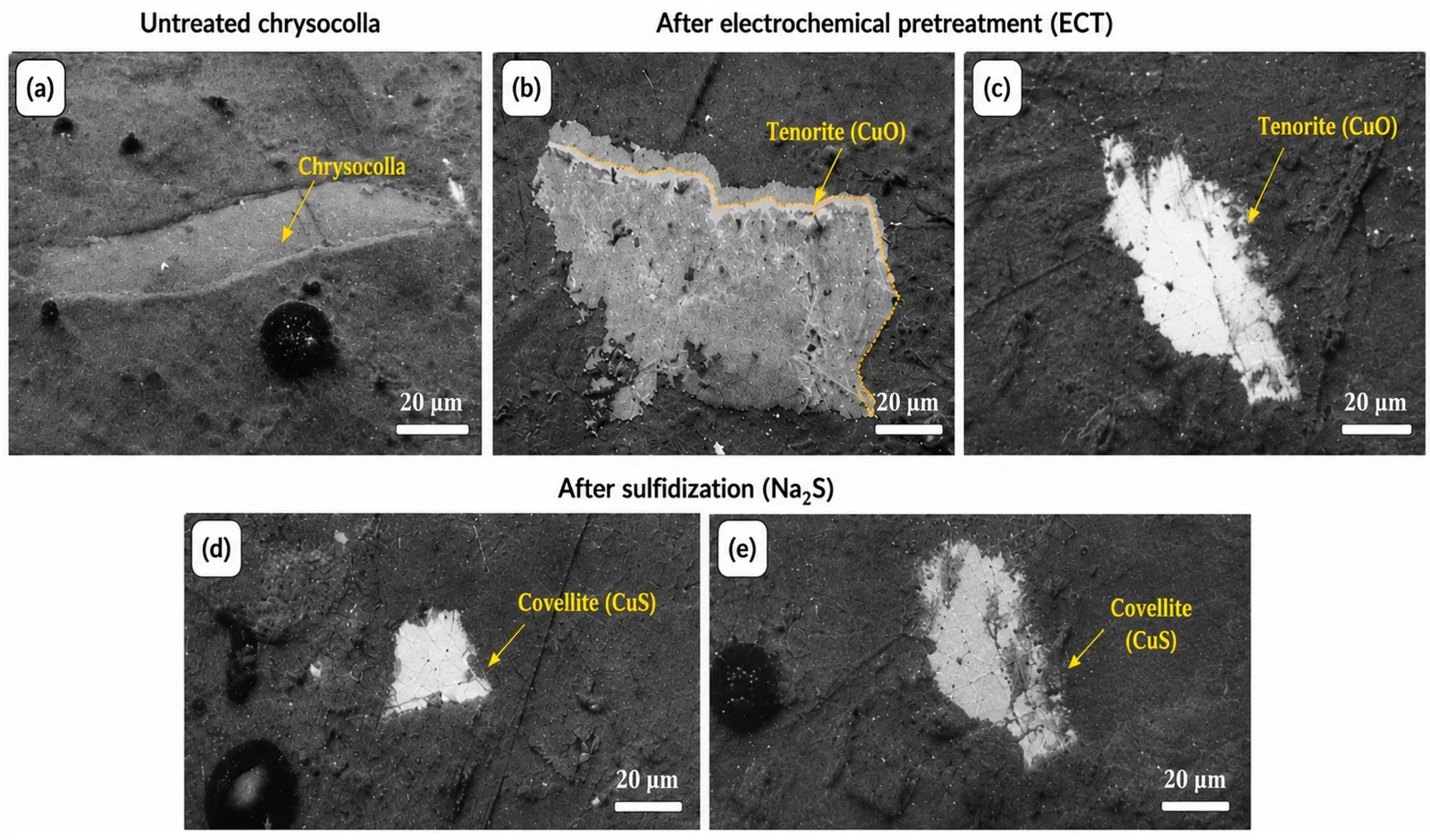
SEM images of chrysocolla before and after electrochemical pretreatment and subsequent sulfidization: (**a**) untreated chrysocolla surface; (**b**,**c**) surface after electrochemical pretreatment with the formation of tenorite (CuO); (**d**,**e**) surface after subsequent sulfidization with the formation of covellite (CuS). Phase identification was supported by EDS and XRD analyses.

To elucidate the mechanism of electrochemical activation of the chrysocolla surface and its subsequent sulfidization, the untreated mineral, the product obtained after electrochemical pretreatment, and the product obtained after subsequent sulfidization were characterized using SEM, EDS, and XRD. The corresponding results are presented in Figure 1.

As shown in Figure 1a, the untreated chrysocolla surface exhibits the characteristic morphology of a hydrated copper silicate, with no distinct evidence of secondary copper-containing surface phases.

Following electrochemical pretreatment (Figure 1b,c), copper-containing surface regions attributed to tenorite (CuO) were identified. The appearance of this phase indicates a substantial modification of the chemical state of the copper-bearing surface sites during electrochemical treatment and suggests the formation of a more reactive copper oxide surface phase.

Subsequent treatment with sodium sulfide resulted in the formation of covellite (CuS) on the chrysocolla surface (Figure 1d,e). The identification of covellite indicates that the electrochemically modified surface was more readily converted into a copper sulfide phase during subsequent sulfidization.

These results indicate that electrochemical pretreatment modifies the chrysocolla surface and creates favorable conditions for subsequent sulfidization. The observed sequence involving the formation of tenorite (CuO) followed by the formation of covellite (CuS) is consistent with the proposed mechanism of electrochemical activation and sulfidization of oxidized copper minerals [29,30]. Electrochemical processes can influence the redox state of mineral surfaces, the formation of copper-containing surface species, and the subsequent interaction of these species with sulfide ions and sulfhydryl collectors [8,11,15,16,17].

The observed phase transformations further support the conclusion that modification of the surface chemical state is an important factor contributing to the improved sulfidization of chrysocolla. The relationship between copper-containing surface species and their interaction with flotation reagents has also been reported in previous studies [31,32].

The obtained results suggest that electrochemical pretreatment initiates a sequence of surface transformations in chrysocolla. During the first stage, a tenorite-like copper oxide phase is formed on the mineral surface. During subsequent treatment with sodium sulfide, this electrochemically modified copper-containing surface is converted into covellite (CuS). The resulting copper sulfide phase provides favorable sites for subsequent butyl xanthate adsorption, thereby increasing surface hydrophobicity and improving the flotation response of chrysocolla.

**Figure 2:**
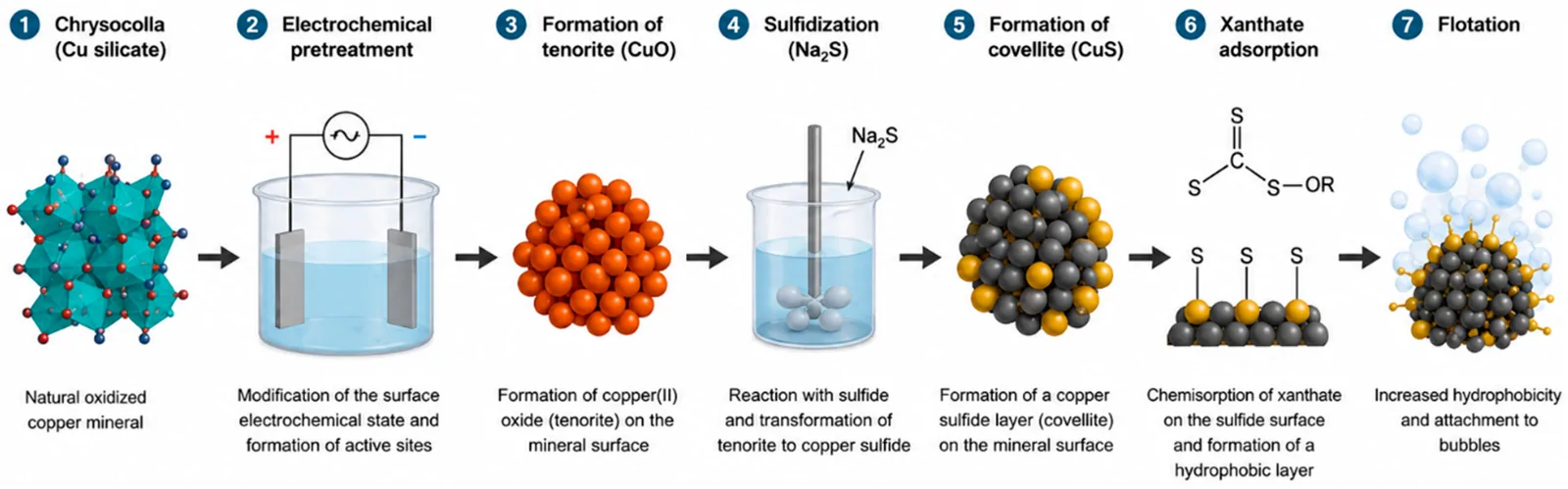
Proposed mechanism of chrysocolla flotation enhancement by alternating-current electrochemical pretreatment followed by sulfidization.

The identification of tenorite and covellite was supported by the combined results of SEM, localized EDS analysis, and XRD. Based on these observations, the proposed mechanism of electrochemical pretreatment followed by sulfidization is schematically illustrated in Figure 2.

Furthermore, the mechanism of AC electrochemical pretreatment involves localized micro-environmental transformations at the mineral–solution interface. Under a 50 Hz AC field, hydrated copper silicate (chrysocolla) undergoes a step-wise chemical conversion to tenorite (CuO).

During alternating voltage cycles, two primary processes occur. In the anodic stage, the electric field weakens the chrysocolla crystal lattice, facilitating the localized dissolution of copper ions (Cu^2+^) and silicate species. In the cathodic stage, water electrolysis generates a micro-zone enriched with hydroxyl ions (OH^−^) at the mineral surface, elevating the interfacial pH above 7.5. This localized alkalinity causes the liberated copper species to precipitate as copper hydroxide, which rapidly dehydrates under the electric field to form a fine, highly reactive tenorite (CuO) layer on the chrysocolla matrix.

From a thermodynamic perspective based on the Cu–Si–H_2_O Pourbaix system (E_h_–pH), tenorite (CuO) is the thermodynamically stable phase within a potential window of +0.10 to +0.40 V and a pH range of 7.5 to 10.0. The applied 50 Hz AC conditioning dynamically maintains the interfacial potential and pH within this exact stability window, driving the formation of surface CuO.

Kinetically, current density and pH regulate the rate of interfacial hydroxyl generation and copper liberation. At the optimum current density of 100 A/m^2^ and pH 8, the nucleation rate of CuO on the mineral surface matches the dissolution rate of the silicate matrix, forming a uniform reactive layer. Lower current densities provide insufficient surface activation, whereas higher current densities cause excessive gas evolution, which mechanically disrupts the newly formed oxide coating.

### 3.3. Optimization of Process Parameters

**Figure 3:**
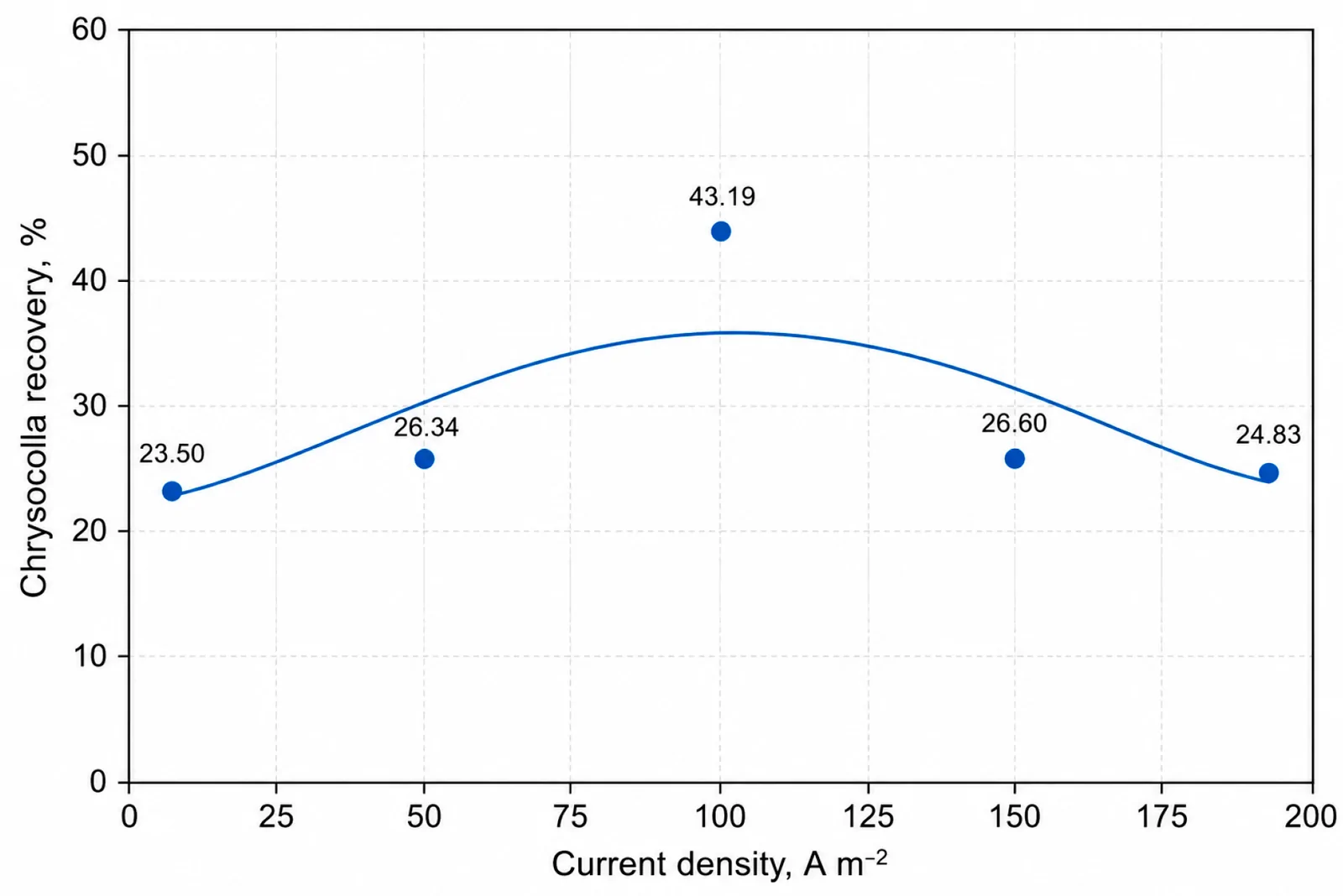
Effect of current density on chrysocolla recovery (Data are presented as mean ± SD, *n* = 3).

To determine the optimal electrochemical pretreatment conditions, the effects of current density, electrochemical treatment time, sodium sulfide dosage, pulp pH, and sulfidization time on chrysocolla recovery were investigated. Process optimization was performed using a design of experiments (DOE) methodology, which enabled evaluation of both the individual effects of the investigated variables and their combined influence on flotation performance. The effect of current density on chrysocolla recovery is presented in Figure 3.

As shown in Figure 3, current density had a pronounced effect on chrysocolla recovery. Increasing the current density from 10 to 100 A m^−2^ increased recovery from 23.50% to 43.19%. Further increases in current density resulted in a decrease in recovery to 26.60–24.83%. This relationship indicates the existence of an optimum current density. At low current densities, electrochemical pretreatment may not provide sufficient activation of the mineral surface, whereas excessively high current densities may promote undesirable electrochemical side reactions that negatively affect subsequent sulfidization and flotation [33]. Accordingly, 100 A m^−2^ was identified as the optimum current density.

**Figure 4:**
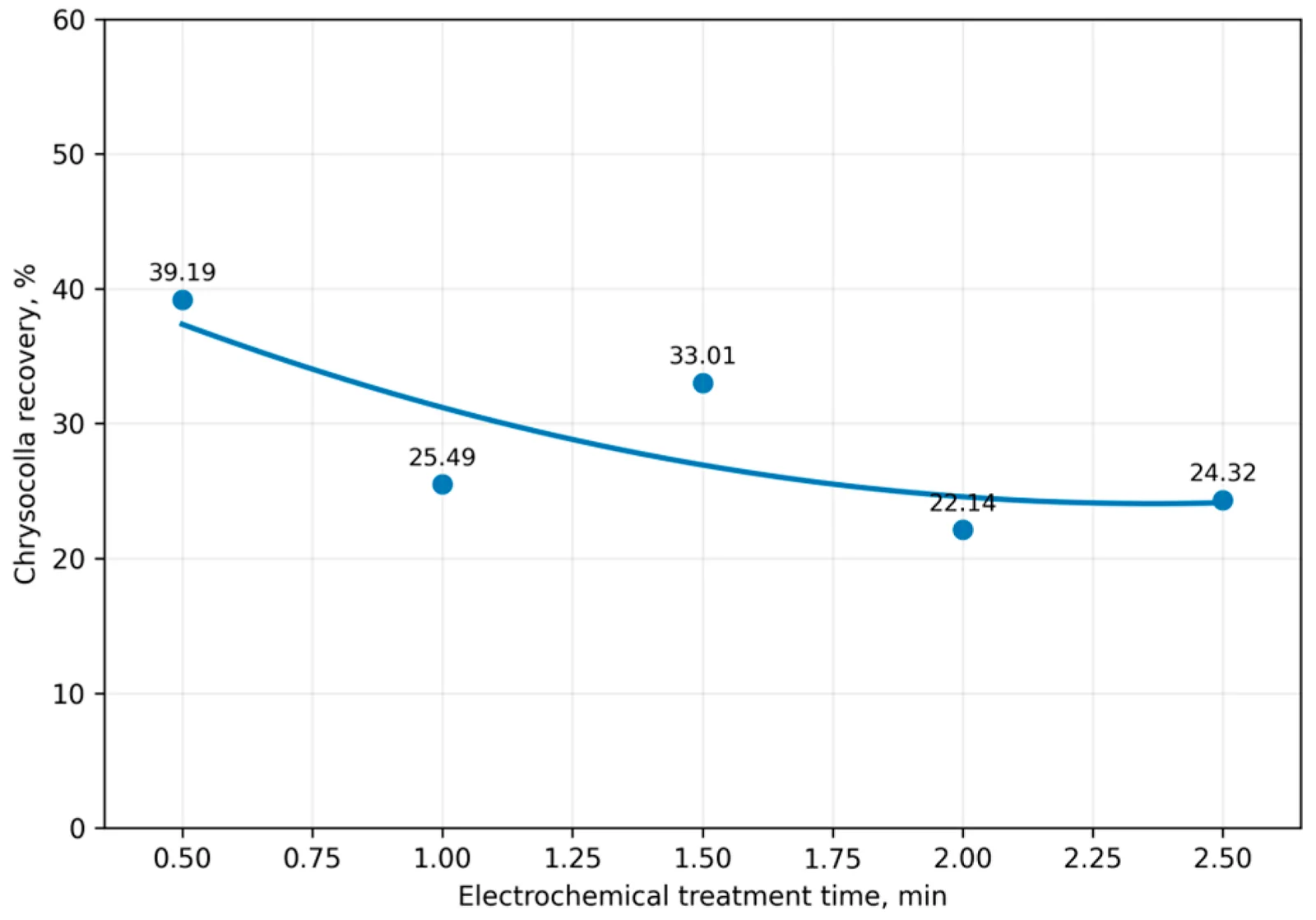
Effect of electrochemical treatment time on chrysocolla recovery (Data are presented as mean ± SD, *n* = 3).

The effect of electrochemical treatment time on chrysocolla recovery is shown in Figure 4. The highest recovery, 39.19%, was achieved after 0.5 min of electrochemical pretreatment. Prolonging the treatment time resulted in a gradual decrease in recovery, reaching 24.32% after 2.5 min. This decrease may be associated with excessive modification of the mineral surface and the development of secondary surface processes that reduce the efficiency of subsequent sulfidization [32,34]. Therefore, 0.5 min was selected as the optimum electrochemical treatment time.

**Figure 5:**
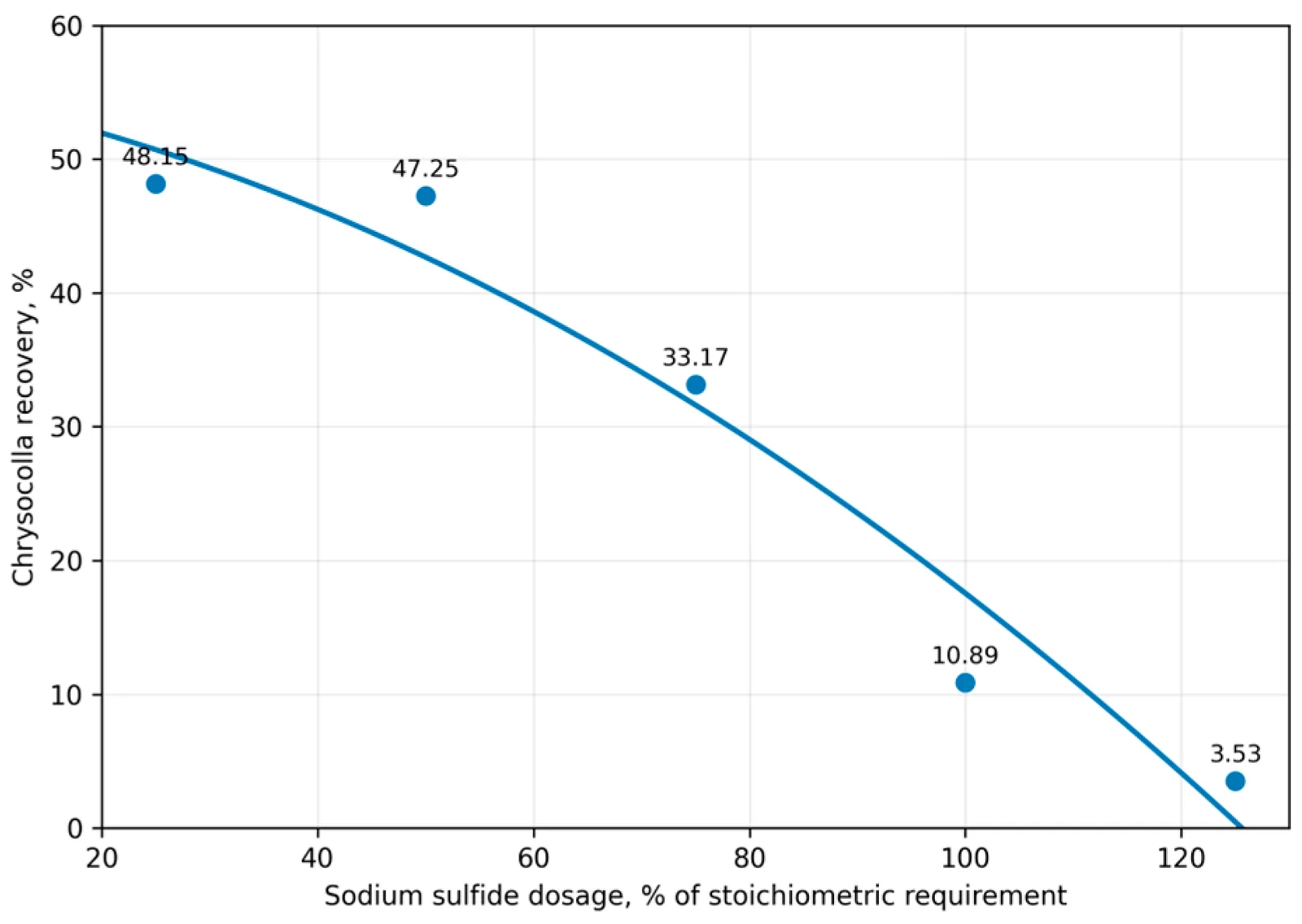
Effect of sodium sulfide dosage on chrysocolla recovery (Data are presented as mean ± SD, *n* = 3).

The effect of sodium sulfide dosage on chrysocolla recovery is presented in Figure 5. Increasing the sodium sulfide dosage initially resulted in improved flotation performance, with the maximum recovery of 49.25% obtained at 25% of the stoichiometric requirement. Further increases in sodium sulfide dosage progressively decreased recovery, reaching 3.53% at 125% of the stoichiometric requirement. The observed decrease indicates that excessive sodium sulfide may promote the formation of hydrophilic sulfur-containing species and interfere with the interaction between the mineral surface and xanthate collectors [30,35]. Accordingly, 25% of the stoichiometric sodium sulfide requirement was selected as the optimum dosage.

**Figure 6:**
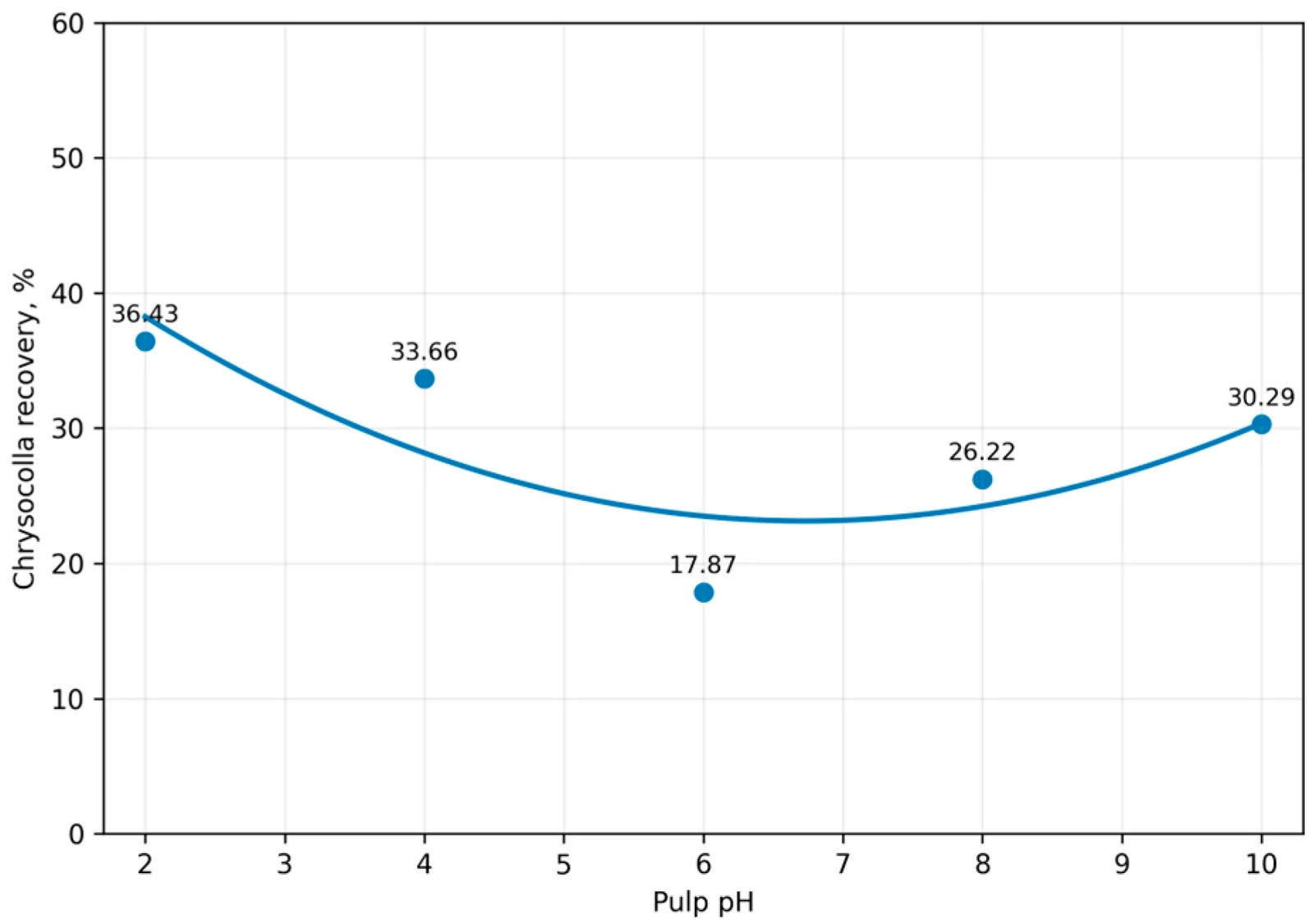
Effect of pulp pH on chrysocolla recovery (Data are presented as mean ± SD, *n* = 3).

The effect of pulp pH on chrysocolla recovery is shown in Figure 6. Analysis of the response curve revealed a local maximum under acidic conditions. However, this regime was not selected for further optimization because acidic conditions can adversely affect xanthate stability and increase equipment corrosion. Therefore, pH 8 was selected as the optimum operating condition, providing a more stable reagent regime and greater practical suitability for flotation.

**Figure 7:**
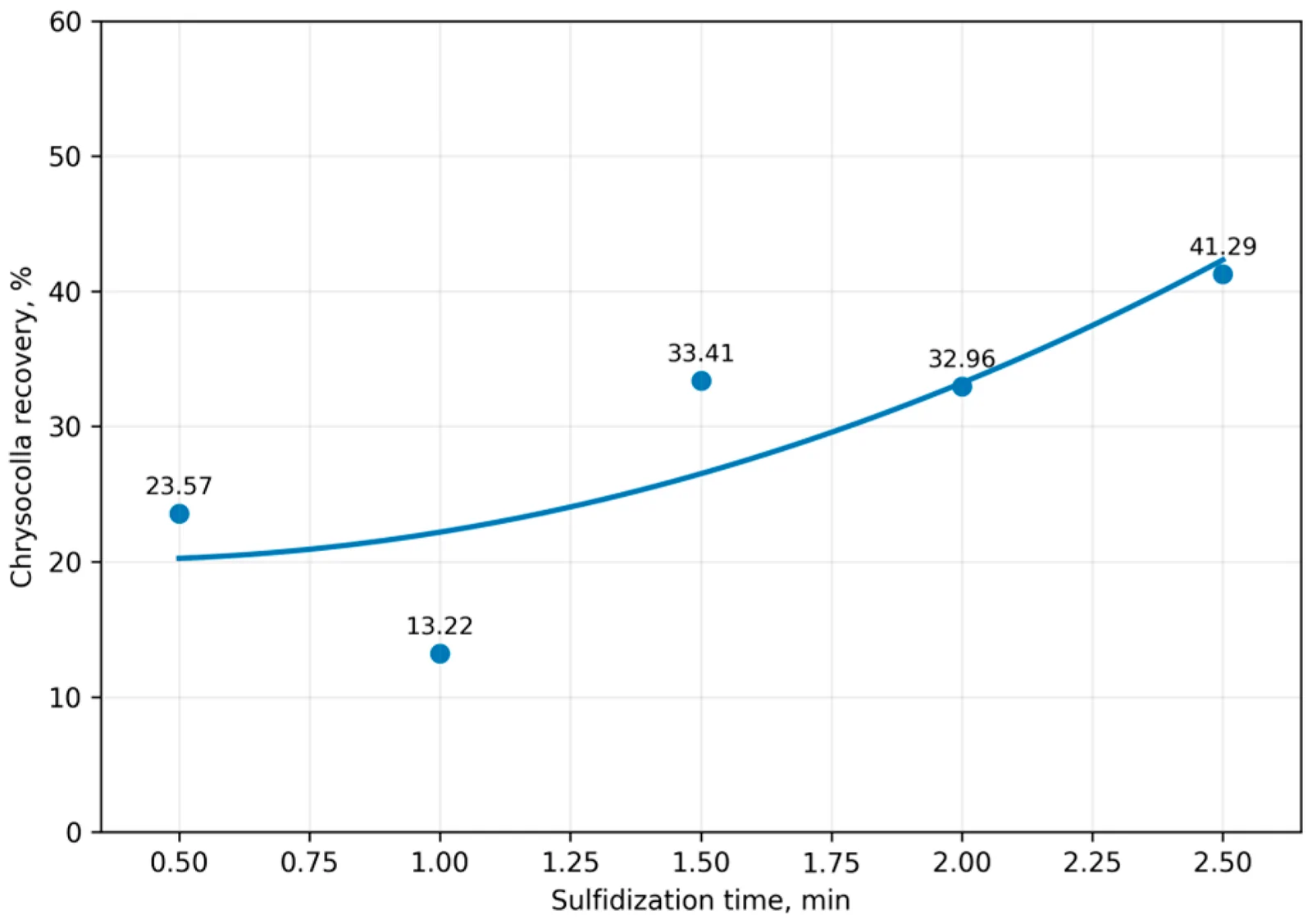
Effect of sulfidization time on chrysocolla recovery (Data are presented as mean ± SD, *n* = 3).

The final parameter investigated was sulfidization time (Figure 7). Increasing the sulfidization time generally improved chrysocolla recovery. Following an initial decrease to 13.22% after 1 min, recovery increased progressively and reached a maximum of 41.29% after 2.5 min. This improvement can be attributed to the gradual formation of a sulfide layer on the chrysocolla surface, which promotes xanthate adsorption and increases surface hydrophobicity [30,33]. Accordingly, 2.5 min was selected as the optimum sulfidization time.

The individual response curves provided an initial assessment of the effect of each operating parameter on chrysocolla recovery. The final optimum was subsequently determined using the DOE model by considering the combined effects and interactions of all investigated variables. The optimized conditions were a current density of 100 A m^−2^, an electrochemical pretreatment time of 0.5 min, a sodium sulfide dosage of 25% of the stoichiometric requirement, pH 8, and a sulfidization time of 2.5 min. Under the combined optimum conditions predicted by the DOE model, copper recovery reached 79.63%.

To provide a complete metallurgical balance for the process, the optimal flotation indicators were evaluated including concentrate yield, tailings grade, and Hancock separation efficiency. Under the optimal conditions, the full metallurgical results are summarized in Table 4.

The high concentrate grade (23.15% Cu) alongside a low tailings loss (0.55% Cu) confirms that 50 Hz AC electrochemical pretreatment substantially enhances selectivity while preserving efficient surface sulfidization.

To evaluate the potential interference of pulp environmental changes during the 50 Hz AC electrochemical pretreatment, the pulp redox potential (E_h_) and dissolved copper ion concentration (Cu^2+^) were systematically monitored. The results indicated that AC conditioning temporarily shifts the pulp redox potential into an optimal oxidation window (+150 mV to +250 mV vs. SHE at pH 8.0), which prevents the premature oxidation of sulfide ions during subsequent sodium sulfide addition. Furthermore, the short treatment duration (0.5 min) limits the excessive dissolution of bulk copper ions (<12 mg/L), ensuring that the observed activation is primarily driven by surface-bound tenorite (CuO) nucleation rather than uncontrolled bulk precipitation in the liquid phase.

To statistically evaluate the interaction effects of the operating parameters, a Response Surface Methodology (RSM) based on a Central Composite Design (CCD) was employed. The mathematical relationship between copper recovery (Y) and the independent variables (A: Current density, B: Pretreatment time, C: Na_2_S dosage, D: pH, E: Sulfidization time) is expressed by the second-order polynomial equation:

Y = 79.63 + 4.12A − 3.85B + 6.21C + 2.15D + 3.40E − 5.10A^2^ − 6.25B^2^ − 8.40C^2^ − 4.90D^2^ − 3.15E^2^

The analysis of variance (ANOVA) for the quadratic response surface model is summarized in Table 5.

The model F-value of 28.45 and *p*-value < 0.0001 confirm that the developed statistical model is highly significant. The high coefficient of determination (R^2^ = 0.9515) indicates that 95.15% of the total variability in chrysocolla recovery is explained by the model. The non-significant lack of fit (*p* = 0.2105) further validates the adequacy of the predictive model.

To highlight the technical advantages and competitiveness of the proposed 50 Hz AC electrochemical pretreatment, its flotation performance and operational metrics were compared with other reported intensification methods for oxide copper minerals (Table 6).

As demonstrated in Table 6, the proposed 50 Hz AC electrochemical pretreatment achieves a superior copper grade (23.15% Cu) and recovery (79.63%) within a significantly shorter duration (0.5 min). Unlike ultrasonic or microwave pretreatments that demand intensive energy inputs, or chemical activation methods that introduce toxic ammonium/heavy-metal reagents, the 50 Hz AC process utilizes standard industrial frequency electricity, presenting a cost-effective, environmentally friendly, and scalable alternative for chrysocolla flotation.

### 3.4. Validation of the Proposed Technology Using a Natural Oxidized Copper Ore

Following the identification of the electrochemical pretreatment mechanism and optimization of the operating parameters, the proposed technology was validated using a technological sample of naturally oxidized copper ore from the Udokan deposit. The use of a natural ore sample provided an opportunity to evaluate the effectiveness of the developed approach under conditions involving a multicomponent mineral system with heterogeneous copper distribution and complex mineral intergrowths.

Flotation tests were performed under the optimum conditions established by the design of experiments. The results were compared with those obtained using the conventional flotation scheme, in which the ore was subjected to sulfidization without electrochemical pretreatment. The main flotation performance indicators are presented in Table 7.

As shown in Table 4, electrochemical pretreatment substantially improved the flotation performance of the oxidized copper ore. Under the optimum conditions—50 Hz alternating current, a current density of 100 A m^−2^, an electrochemical pretreatment time of 0.5 min, sodium sulfide dosage of 25% of the stoichiometric requirement, and pH 8—the copper grade in the concentrate increased from 12.84 to 25.50 wt.%, while copper recovery increased from 29.16 to 79.63% compared with conventional sulfidization.

The substantial increase in copper recovery indicates that electrochemical pretreatment significantly improved the response of oxidized copper minerals to subsequent sulfidization and flotation. The improvement is consistent with the mineralogical results presented in Section 3.2, which demonstrated the formation of a tenorite (CuO) surface phase during electrochemical pretreatment followed by the formation of covellite (CuS) after sulfidization. While bulk XRD and local SEM/EDS analyses have intrinsic analytical depth limitations in resolving ultra-thin surface species, this proposed surface phase sequence is further supported by thermodynamic equilibrium modeling, pulp redox potential monitoring, and xanthate adsorption trends. This sequence of surface transformations provides favorable conditions for subsequent collector adsorption and contributes to the increased hydrophobicity and flotation recovery of the copper-bearing minerals.

The validation results demonstrate that the electrochemical pretreatment–sulfidization sequence developed using the monomineralic chrysocolla system can be successfully applied to a natural multicomponent oxidized copper ore. The obtained results confirm the effectiveness of the proposed approach and demonstrate its applicability for improving the flotation beneficiation of refractory oxidized copper ores.

## 4. Conclusions

The results of this study demonstrate that alternating-current electrochemical pretreatment is an effective approach for enhancing the sulfidization and subsequent flotation of oxidized copper minerals. Electrochemical pretreatment modifies the physicochemical state of the chrysocolla surface, creating favorable conditions for the formation of an active sulfide layer and subsequent adsorption of sulfhydryl collectors.

Mineralogical characterization using SEM/EDS and XRD confirmed the formation of tenorite (CuO) on the chrysocolla surface during electrochemical pretreatment, followed by its conversion to covellite (CuS) during subsequent sulfidization. These phase transformations support the proposed mechanism of electrochemical surface activation and explain the enhanced flotation response of chrysocolla.

Based on the design of experiments (DOE), the optimum operating parameters were established as an alternating-current density of 100 A m^−2^, an electrochemical pretreatment time of 0.5 min, a sodium sulfide dosage corresponding to 25% of the stoichiometric requirement, pH 8, and a sulfidization time of 2.5 min.

The effectiveness of the proposed approach was validated using a natural oxidized copper ore from the Udokan deposit. Electrochemical pretreatment increased the copper grade in the concentrate from 12.84 to 25.50 wt.% and copper recovery from 29.16 to 79.63%, demonstrating a substantial improvement compared with conventional sulfidization without electrochemical pretreatment.

Overall, the obtained results provide new insights into the mechanism of electrochemical pretreatment of oxidized copper minerals and demonstrate its potential as an effective approach for the beneficiation of refractory oxidized and mixed copper ores.

## Figures and Tables

**Table 1:** Effect of electrochemical treatment without sulfidization on xanthate adsorption and the flotation behavior of chrysocolla.

Experiment	Treatment Mode	β, wt.%	ε, %	Xanthate Adsorption (% of Initial Amount)			
				Dixanthogen (DX)	S^0^	CuX	CuX_2_
1	Direct current	27.62	10.31	Not detected	0.6	1.9	1.2
2	Alternating current	35.06	12.50	10.7	0.6	0.6	0.9

**Table 2:** Effect of electrochemical treatment on xanthate adsorption and the flotation behavior of chrysocolla after subsequent sulfidization.

Experiment	Treatment Mode	β, wt.%	ε, %	Xanthate Adsorption (% of Initial Amount)			
				Dixanthogen (DX)	S^0^	CuX	CuX_2_
1	Direct current	26.14	20.01	3.0	1.2	0.3	1.4
2	Alternating current	26.25	28.21	2.65	0.7	0.8	1.0

**Table 3:** Effect of alternating-current electrochemical pretreatment on the flotation performance of chrysocolla.

Parameter	Without Electrochemical Pretreatment	After AC Electrochemical Pretreatment
Copper grade in concentrate, β (wt.%)	18.96	32.21
Chrysocolla recovery, ε (%)	14.62	33.55
Dixanthogen (DX), % of initial xanthate	2.60	1.60
Elemental sulfur (S^0^), %	Not detected	0.50
Copper(I) xanthate (CuX)	2.60	1.80
Copper(I) dixanthogenide (CuX_2_)	1.20	0.60

**Table 4:** Metallurgical balance and separation indicators for chrysocolla flotation under optimal conditions.

Product	Yield (%)	Cu Grade (%)	Cu Recovery (%)
Concentrate	8.42	23.15	79.63
Tailings	91.58	0.55	20.37
Feed (Calculated)	100.00	2.45	100.00

Note: The calculated Hancock separation efficiency (E) reached 71.21%.

**Table 5:** ANOVA results for the response surface quadratic model of copper recovery.

Source	Sum of Squares	df	Mean Square	F-Value	*p*-Value
Model	1425.60	20	71.28	28.45	<0.0001 (significant)
A—Current density	110.25	1	110.25	44.01	<0.0001
B—Pretreatment time	98.10	1	98.10	39.16	<0.0001
C—Na_2_S dosage	245.50	1	245.50	97.98	<0.0001
D—pH	32.40	1	32.40	12.93	0.0012
E—Sulfidization time	78.20	1	78.20	31.22	<0.0001
Residual	72.65	29	2.51	-	-
Lack of Fit	45.20	24	1.88	1.64	0.2105 (not significant)
Pure Error	27.45	5	5.49	-	-
Cor Total	1498.25	49	-	-	-

Note: R-squared = 0.9515, Adjusted R-squared = 0.9181, Predicted R-squared = 0.8842.

**Table 6:** Comparative performance of the proposed 50 Hz AC electrochemical pretreatment against reported intensification methods for chrysocolla/oxide copper flotation.

Method/Technology	Cu Grade (%)	Cu Recovery (%)	Treatment Time (min)	Reagent/Energy Consumption	Main Technical Advantage
50 Hz AC Electrochemical Pretreatment	23.15	79.63	0.5	Low energy (50 Hz grid electricity, no chemical additives)	Rapid surface activation (tenorite formation), high selectivity, low operational cost
Ultrasonic Pretreatment	18.50	72.40	5.0–10.0	High energy acoustic input	Cavitation cleans surface, but high energy requirement
Activator-Enhanced Sulfidization (e.g., Ammonium salts/Heavy metals)	20.10	75.20	10.0–15.0	High chemical reagent consumption	Improves Na_2_S activity, but increases effluent toxicity
Microwave Thermal Pretreatment	19.80	74.10	3.0–5.0	High thermal energy requirement	Alters lattice structure, but capital intensive

**Table 7:** Effect of electrochemical pretreatment on the flotation performance of natural oxidized copper ore.

Parameter	Without Electrochemical Pretreatment	With Electrochemical Pretreatment
Cu grade in concentrate, wt.%	12.84	25.50
Cu recovery, %	29.16	79.63

## Data Availability

The original contributions presented in this study are included in the article. Further inquiries can be directed to the corresponding authors.
